# (*E*)-Methyl 3-(10-bromo­anthracen-9-yl)acrylate

**DOI:** 10.1107/S1600536813016905

**Published:** 2013-06-22

**Authors:** Bernhard Bugenhagen, Yosef Al Jasem, Bassam al Hindawi, Nathir Al Rawashdeh, Thies Thiemann

**Affiliations:** a Institute of Inorganic Chemistry, University of Hamburg, Hamburg, Germany; b Department of Chemical Engineering, United Arab Emirates University, AL Ain, Abu Dhabi, United Arab Emirates; c Department of Chemistry, United Arab Emirates University, AL Ain, Abu Dhabi, United Arab Emirates

## Abstract

In the title mol­ecule, C_18_H_13_BrO_2_, the anthracene unit forms an angle of 46.91 (2)° with the mean plane of the methyl acrylate moiety. In the crystal, the mol­ecules arrange themselves into strands parallel to [010] and, due to the crystal symmetry, there are eight strands crossing the unit cell. In each strand, mol­ecules form short C—H⋯O and C—H⋯π contacts and have their anthracene groups parallel to each other. Neighboring strands, related by a *c*-glide operation, are connected *via* C—H⋯O inter­actions and form a layer parallel to (100). The arrangement of the acrylate and anthracene groups in the crystal do not allow for [2 + 2] or [4 + 4] cyclo­addition.

## Related literature

For an analogous preparation of the title compound, see: Bugenhagen *et al.* (2013 ▶); Nguyen & Weizman (2007 ▶). For crystal structures of photodimerizable aryl-enes, see: Vishnumurthy *et al.* (2002 ▶); Mascitti & Corey (2006 ▶); Sonoda (2011 ▶); Schmidt (1964 ▶). For the photodimerization of anthracenes in the crystal, see: Schmidt (1971 ▶); Ihmels *et al.* (2000 ▶). For the X-ray crystal structure of a non-planar bromo­anthracene, see: Barkhuizen *et al.* (2004 ▶).
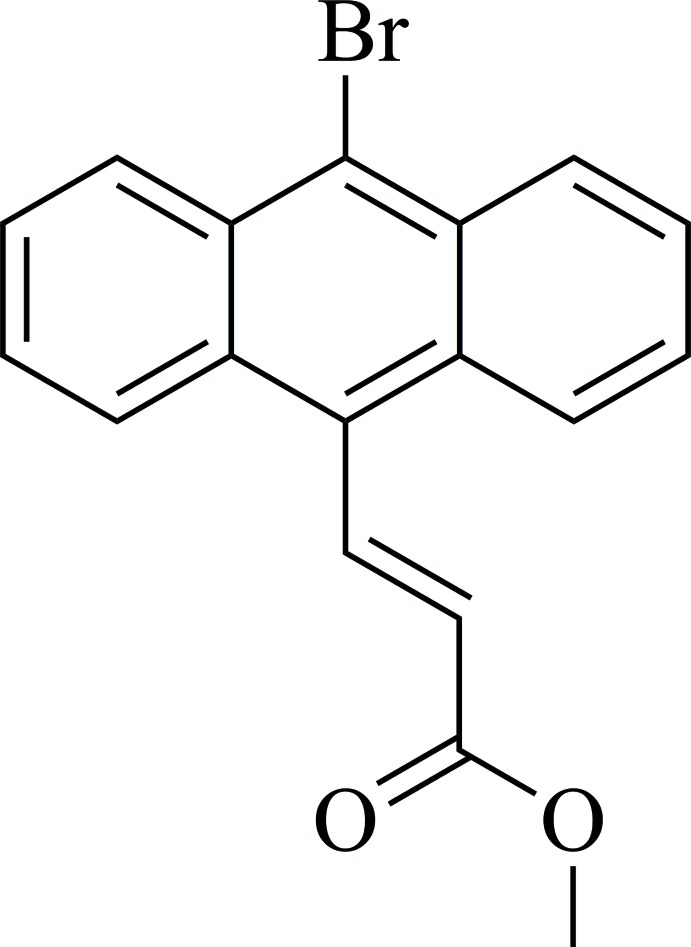


## Experimental

### 

#### Crystal data

C_18_H_13_BrO_2_
*M* *_r_* = 341.19
Orthorhombic, 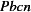

*a* = 40.5848 (4) Å
*b* = 5.32093 (5) Å
*c* = 13.0710 (1) Å
*V* = 2822.67 (5) Å^3^
*Z* = 8
Cu *K*α radiation
μ = 3.98 mm^−1^
*T* = 100 K
0.21 × 0.19 × 0.13 mm

#### Data collection

Oxford Diffraction SuperNova, Dual, Cu at zero, Atlas diffractometer
Absorption correction: analytical (*CrysAlis PRO*; Agilent, 2013 ▶) *T*_min_ = 0.891, *T*_max_ = 0.924
25313 measured reflections
2961 independent reflections
2900 reflections with *I* > 2σ(*I*)
*R*_int_ = 0.022

#### Refinement

*R*[*F* ^2^ > 2σ(*F* ^2^)] = 0.026
*wR*(*F* ^2^) = 0.066
*S* = 1.12
2961 reflections
191 parameters
H-atom parameters constrained
Δρ_max_ = 0.31 e Å^−3^
Δρ_min_ = −0.44 e Å^−3^

### 

Data collection: *CrysAlis PRO* (Agilent, 2013 ▶); cell refinement: *CrysAlis PRO*; data reduction: *CrysAlis PRO*; program(s) used to solve structure: *SHELXS97* (Sheldrick, 2008 ▶); program(s) used to refine structure: *SHELXL97* (Sheldrick, 2008 ▶) within *OLEX2* (Dolomanov *et al.*, 2009 ▶); molecular graphics: *PLATON* (Spek, 2009 ▶) and *Mercury* (Macrae *et al.*, 2008 ▶); software used to prepare material for publication: *SHELXL97* and *PLATON*.

## Supplementary Material

Crystal structure: contains datablock(s) global, I. DOI: 10.1107/S1600536813016905/gk2574sup1.cif

Structure factors: contains datablock(s) I. DOI: 10.1107/S1600536813016905/gk2574Isup2.hkl

Supplementary material file. DOI: 10.1107/S1600536813016905/gk2574Isup3.cml

Additional supplementary materials:  crystallographic information; 3D view; checkCIF report

## Figures and Tables

**Table 1:** Hydrogen-bond geometry (Å, °) *Cg*1 is the centroid of the C2–C7 ring.

*D*—H⋯*A*	*D*—H	H⋯*A*	*D*⋯*A*	*D*—H⋯*A*
C15—H15⋯*Cg*1^i^	0.93	2.91 (2)	3.5055 (19)	123
C16—H16⋯O2^ii^	0.93	2.40	3.315 (3)	170
C5—H5⋯O2^iii^	0.93	2.53	3.372 (2)	150

Symmetry codes: (i) 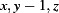; (ii) 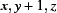; (iii) 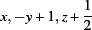.

**Fig. 1.**
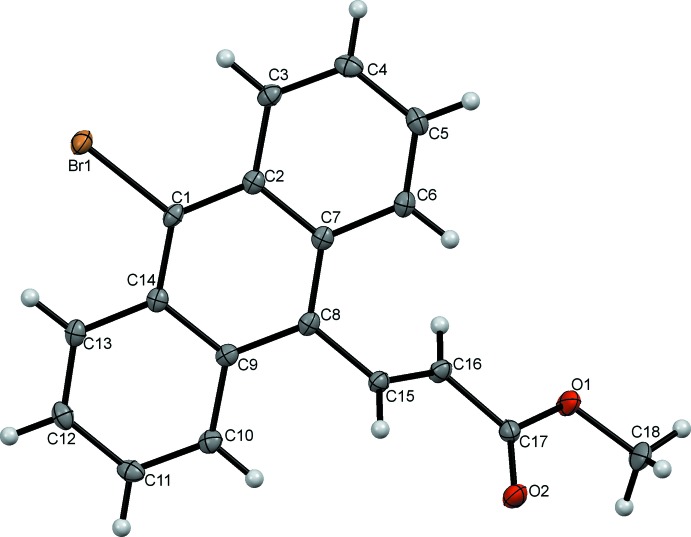
A view of the title molecule with the atom-numbering scheme. Displacement ellipsoids are shown at the 50% probability level.

**Fig. 2.**
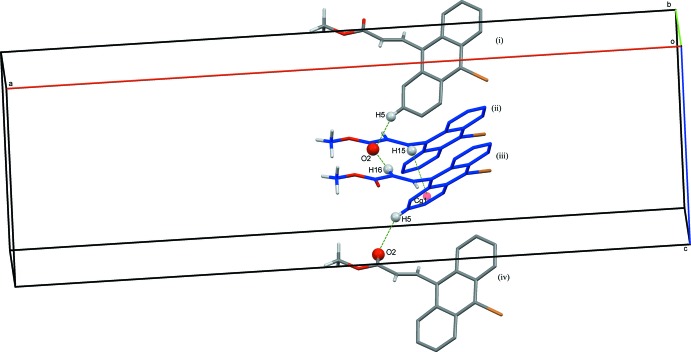
The close contacts C—H···O and C—H···π between two molecules (colored in blue) in one strand, in addition to the C—H···O interaction between molecules of neighboring strands (related by c-glide operation). [Symmetry codes: (i) x,2 - y,-1/2 + z; (ii) x,1 + y,z; (iii) x,y,z; (iv) x,1 - y,1/2 + z]

**Fig. 3.**
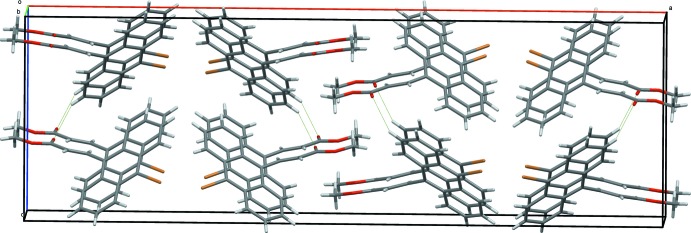
The eight strands crossing the unit cell and propagating along the b axis with C—H···O interaction between neighboring strands shown as dashed lines.

## supplementary crystallographic information

### Comment

In our interest in [2 + 2]-photocycloaddition of anthracene derivatives in the crystal (Sonoda, 2011; Schmidt, 1964), the authors grew single crystals of the title compound. The bromo atom (C2—Br1—C14 plane) deviates from the averaged plane of the anthracenyl unit (C1—C14) at an angle of 3.66 (2)°. This unusual deviation (Barkhuizen *et al.*, 2004) may be due to the forced closeness of Br1 to the π system (C9—C14) of the underlying anthracenyl unit. Eight strands of molecules of the title compound cross the unit cell and propagate along [010] (Figure 3). The neighboring strands that are related by c-glide operation are connected *via* C5—H5···O2 interaction (Table 1) and form a layer parallel to (100). The close contacts C16—H16···O2 and C15—H15···*Cg*1 (π) (Table 1) link the neighboring molecules within each strand (Figure 2). The average plane of an anthracenyl unit of a molecule in one strand forms an angle of 78.37 (2)° with an anthracenyl unit of a molecule in the neighboring strand.While the anthracenyl units of the molecules in one strand are parallel to each other and exhibit an off-set that would be beneficial for π-π interaction, they are too far apart (distance between the averaged planes of the respective anthracenyl units: 3.362 (2) Å) to exhibit a strong π-π interaction, in contrast with the non-brominated parent compound (Bugenhagen *et al.*, 2013).

The closest distance between double bonds of two molecules, which are molecules in one strand, is 5.321 (3) Å. Although this distance is smaller than the smallest distance found in the non-brominated parent compound (5.549 (3) Å) (Bugenhagen *et al.*, 2013), it is larger than in many of those found for aryl-enes that undergo [2 + 2]-photodimerization readily (Vishnumurthy *et al.* 2002; Mascitti *et al.* 2006). The anthracenyl units themselves, while aligned parallel to each other in one strand, are off-set to each other and are much further apart (5.321 (3) Å for C1—C1 and C8—C8) than in anthracenes (less than 4.2 Å) that have been reported to undergo [4 + 4]-photodimerization in the crystal (Schmidt, 1971; Ihmels *et al.*, 2000).

### Experimental

Methyl (*E*)-3-[10-bromoanthran-9-yl]-2-propenoate: to a solution of (*E*)-3-[10-bromoanthran-9-yl]-2-propenoic acid (875 mg, 2.68 mmol) in 1,2-dichloroethane (10 ml) was given an excess of thionyl chloride (1.0 g, 8.54 mmol), and the resulting mixture was kept at 70°C for 1.5 h. Thereafter, the ensuing solution was concentrated *in vacuo*. Thereafter, a solution of methanol (7 ml) in 1,2-dichloroethane (10 ml) was added, and the mixture was stirred at rt for 10 h. Then, it was concentrated *in vacuo*. The residue was taken up in dichloromethane (20 ml), extracted with water (2 *X* 10 ml), and dried over MgSO_4_. The solution was concentrated *in vacuo*, and the residue was subjected to column chromatography on silica gel (benzene-hexane 1:1) to give the title compound (847 mg, 93%) as a yellow solid; mp. 425 K; IR (KBr/cm^-1^) *n*_max_ 3066, 3021, 2950, 1711 (CO), 1631, 1436, 1338, 1315, 1254, 1203, 1180, 999, 900, 762, 749, 726; *d*_H_ (400 MHz, CDCl_3_) 3.92 (3*H*, s, OCH_3_), 6.38 (1*H*, d, ^3^*J* = 16.0 Hz), 7.51 – 7.55 (2*H*, m), 7.59 – 7.63 (2*H*, m), 8.20 (2*H*, d, ^3^*J* = 8.8 Hz), 8.55 (1*H*, d, ^3^*J* = 16.0 Hz), 8.57 (2*H*, d, ^3^*J* = 7.6 Hz); *d*_C_ (100.5 MHz, CDCl_3_) 52.0 (OCH_3_), 124.5 (C_quat_), 125.7 (2 C, CH), 126.4 (2 C, CH), 127.2 (2 C, CH), 127.7 (CH), 128.4 (2 C, CH), 129.8 (2 C, C_quat_), 130.1 (C_quat_), 130.2 (2 C, C_quat_), 142.0 (CH), 166.6 (C_quat_, CO).

### Refinement

All carbon-bound hydrogen atoms were placed in calculated positions with C—H distances of 0.93 - 0.96 Å and refined as riding with *U*_iso_(H) =*xU*_eq_(C), where *x* = 1.5 for methyl and *x* = 1.2 for all other H-atoms.

### Crystal data

**Table:**

C_18_H_13_BrO_2_	*D*_x_ = 1.606 Mg m^−^^3^
*M**_r_* = 341.19	Melting point: 425 K
Orthorhombic, *P**b**c**n*	Cu *K*α radiation, λ = 1.5418 Å
*a* = 40.5848 (4) Å	Cell parameters from 13502 reflections
*b* = 5.32093 (5) Å	θ = 4.3–76.3°
*c* = 13.0710 (1) Å	µ = 3.98 mm^−^^1^
*V* = 2822.67 (5) Å^3^	*T* = 100 K
*Z* = 8	Block, light yellow
*F*(000) = 1376	0.21 × 0.19 × 0.13 mm

### Data collection

**Table:**

Oxford Diffraction SuperNova, Dual, Cu at zero, Atlas diffractometer	2961 independent reflections
Radiation source: SuperNova (Cu) X-ray Source	2900 reflections with *I* > 2σ(*I*)
Mirror monochromator	*R*_int_ = 0.022
Detector resolution: 10.4127 pixels mm^-1^	θ_max_ = 76.3°, θ_min_ = 4.4°
ω scans	*h* = −50→44
Absorption correction: analytical (*CrysAlis PRO*; Agilent, 2013)	*k* = −5→6
*T*_min_ = 0.891, *T*_max_ = 0.924	*l* = −15→16
25313 measured reflections	

### Refinement

**Table:**

Refinement on *F*^2^	Primary atom site location: structure-invariant direct methods
Least-squares matrix: full	Hydrogen site location: inferred from neighbouring sites
*R*[*F*^2^ > 2σ(*F*^2^)] = 0.026	H-atom parameters constrained
*wR*(*F*^2^) = 0.066	*w* = 1/[σ^2^(*F*_o_^2^) + (0.0253*P*)^2^ + 4.4132*P*] where *P* = (*F*_o_^2^ + 2*F*_c_^2^)/3
*S* = 1.12	(Δ/σ)_max_ = 0.003
2961 reflections	Δρ_max_ = 0.31 e Å^−^^3^
191 parameters	Δρ_min_ = −0.44 e Å^−^^3^
0 restraints	

### Special details

**Table:**

Geometry. All e.s.d.'s (except the e.s.d. in the dihedral angle between two l.s. planes) are estimated using the full covariance matrix. The cell e.s.d.'s are taken into account individually in the estimation of e.s.d.'s in distances, angles and torsion angles; correlations between e.s.d.'s in cell parameters are only used when they are defined by crystal symmetry. An approximate (isotropic) treatment of cell e.s.d.'s is used for estimating e.s.d.'s involving l.s. planes.

### Fractional atomic coordinates and isotropic or equivalent isotropic displacement parameters (Å^2^)

**Table:**

	*x*	*y*	*z*	*U*_iso_*/*U*_eq_	
Br1	0.28462 (2)	1.11853 (4)	0.77185 (2)	0.01637 (8)	
C1	0.32116 (4)	0.9076 (3)	0.73973 (14)	0.0125 (4)	
C10	0.34022 (5)	0.3729 (3)	0.56492 (14)	0.0143 (4)	
C11	0.31165 (5)	0.3554 (4)	0.51014 (14)	0.0159 (4)	
C12	0.28558 (4)	0.5263 (4)	0.52784 (14)	0.0157 (4)	
C13	0.28813 (4)	0.7045 (4)	0.60248 (14)	0.0141 (4)	
C14	0.31732 (4)	0.7266 (3)	0.66306 (13)	0.0121 (3)	
C15	0.40212 (4)	0.4163 (3)	0.67191 (14)	0.0132 (4)	
C16	0.43266 (4)	0.4937 (4)	0.65111 (14)	0.0145 (4)	
C17	0.45923 (4)	0.3109 (4)	0.63028 (14)	0.0147 (4)	
C18	0.51645 (5)	0.2672 (4)	0.60385 (19)	0.0263 (5)	
C2	0.35047 (4)	0.9392 (3)	0.79499 (14)	0.0122 (3)	
C3	0.35474 (5)	1.1300 (3)	0.87078 (14)	0.0144 (4)	
C4	0.38315 (5)	1.1478 (4)	0.92569 (14)	0.0165 (4)	
C5	0.40887 (4)	0.9717 (4)	0.91069 (14)	0.0160 (4)	
C6	0.40616 (4)	0.7915 (4)	0.83732 (14)	0.0148 (4)	
C7	0.37738 (4)	0.7705 (3)	0.77405 (13)	0.0119 (3)	
C8	0.37435 (4)	0.5854 (3)	0.69716 (14)	0.0120 (3)	
C9	0.34444 (4)	0.5594 (3)	0.64271 (14)	0.0120 (3)	
H10	0.3573	0.2613	0.5514	0.017*	
H11	0.3093	0.2304	0.4609	0.019*	
H12	0.2666	0.5171	0.4883	0.019*	
H13	0.2706	0.8133	0.6141	0.017*	
H15	0.3980	0.2444	0.6704	0.016*	
H16	0.4373	0.6648	0.6497	0.017*	
H18A	0.5146	0.1947	0.5368	0.039*	
H18B	0.5169	0.1357	0.6541	0.039*	
H18C	0.5364	0.3639	0.6081	0.039*	
H3	0.3378	1.2440	0.8828	0.017*	
H4	0.3857	1.2763	0.9733	0.020*	
H5	0.4277	0.9793	0.9512	0.019*	
H6	0.4234	0.6788	0.8279	0.018*	
O1	0.48841 (3)	0.4289 (3)	0.62263 (12)	0.0218 (3)	
O2	0.45611 (3)	0.0860 (3)	0.62159 (11)	0.0188 (3)	

### Atomic displacement parameters (Å^2^)

**Table:**

	*U*^11^	*U*^22^	*U*^33^	*U*^12^	*U*^13^	*U*^23^
Br1	0.01322 (11)	0.01739 (12)	0.01851 (12)	0.00440 (7)	0.00145 (7)	−0.00092 (7)
C1	0.0115 (8)	0.0126 (8)	0.0134 (8)	0.0022 (7)	0.0032 (7)	0.0026 (7)
C10	0.0131 (8)	0.0149 (9)	0.0150 (8)	−0.0007 (7)	0.0037 (7)	−0.0002 (7)
C11	0.0174 (9)	0.0177 (9)	0.0126 (8)	−0.0038 (7)	0.0028 (7)	−0.0025 (7)
C12	0.0129 (8)	0.0203 (10)	0.0140 (9)	−0.0035 (7)	−0.0003 (7)	0.0027 (8)
C13	0.0115 (8)	0.0151 (9)	0.0155 (9)	0.0000 (7)	0.0014 (7)	0.0039 (8)
C14	0.0111 (8)	0.0130 (8)	0.0122 (8)	−0.0004 (7)	0.0020 (6)	0.0030 (7)
C15	0.0141 (8)	0.0119 (8)	0.0137 (8)	0.0019 (7)	−0.0009 (7)	−0.0001 (7)
C16	0.0145 (8)	0.0111 (8)	0.0180 (9)	0.0009 (7)	0.0015 (7)	0.0005 (7)
C17	0.0118 (8)	0.0175 (9)	0.0149 (8)	−0.0002 (7)	−0.0009 (7)	−0.0021 (7)
C18	0.0111 (9)	0.0307 (12)	0.0371 (12)	0.0045 (8)	0.0014 (8)	−0.0118 (10)
C2	0.0119 (8)	0.0121 (8)	0.0126 (8)	−0.0005 (7)	0.0024 (7)	0.0030 (7)
C3	0.0144 (8)	0.0137 (9)	0.0151 (9)	0.0008 (7)	0.0033 (7)	0.0000 (7)
C4	0.0189 (9)	0.0173 (9)	0.0134 (8)	−0.0037 (7)	0.0027 (7)	−0.0017 (7)
C5	0.0124 (8)	0.0207 (10)	0.0148 (8)	−0.0030 (7)	−0.0016 (7)	0.0015 (8)
C6	0.0117 (8)	0.0161 (9)	0.0167 (9)	0.0009 (7)	0.0005 (7)	0.0022 (7)
C7	0.0117 (8)	0.0113 (8)	0.0128 (8)	−0.0008 (7)	0.0019 (6)	0.0031 (7)
C8	0.0114 (8)	0.0109 (8)	0.0138 (8)	0.0005 (7)	0.0021 (7)	0.0032 (7)
C9	0.0119 (8)	0.0113 (8)	0.0128 (8)	−0.0009 (7)	0.0031 (6)	0.0011 (7)
O1	0.0098 (6)	0.0199 (7)	0.0356 (8)	−0.0004 (5)	0.0041 (6)	−0.0088 (6)
O2	0.0162 (6)	0.0136 (7)	0.0266 (7)	0.0024 (5)	0.0007 (6)	−0.0016 (6)

### Geometric parameters (Å, º)

**Table:**

C1—Br1	1.9068 (18)	C18—H18C	0.9600
C1—C14	1.398 (3)	C18—H18B	0.9600
C1—C2	1.402 (3)	C18—H18A	0.9600
C10—C11	1.366 (3)	C2—C7	1.440 (2)
C10—H10	0.9300	C2—C3	1.429 (3)
C11—C12	1.414 (3)	C3—C4	1.361 (3)
C11—H11	0.9300	C3—H3	0.9300
C12—C13	1.364 (3)	C4—C5	1.416 (3)
C12—H12	0.9300	C4—H4	0.9300
C13—C14	1.430 (2)	C5—C6	1.361 (3)
C13—H13	0.9300	C5—H5	0.9300
C15—C16	1.334 (3)	C6—C7	1.435 (2)
C15—H15	0.9300	C6—H6	0.9300
C16—C17	1.478 (3)	C7—C8	1.413 (3)
C16—H16	0.9300	C8—C15	1.479 (2)
C17—O2	1.208 (2)	C8—C9	1.414 (2)
C17—O1	1.344 (2)	C9—C14	1.440 (2)
C18—O1	1.448 (2)	C9—C10	1.431 (3)
			
C2—C1—Br1	118.41 (14)	C11—C10—H10	119.3
C14—C1—C2	123.12 (17)	C10—C11—H11	119.8
C14—C1—Br1	118.46 (14)	C10—C11—C12	120.37 (18)
C1—C2—C3	123.06 (17)	C12—C11—H11	119.8
C1—C2—C7	118.09 (17)	C11—C12—H12	119.8
C3—C2—C7	118.85 (17)	C13—C12—C11	120.48 (17)
C2—C3—H3	119.4	C13—C12—H12	119.8
C4—C3—C2	121.20 (18)	C12—C13—H13	119.5
C4—C3—H3	119.4	C12—C13—C14	121.07 (17)
C3—C4—H4	119.8	C14—C13—H13	119.5
C3—C4—C5	120.32 (18)	C1—C14—C9	118.22 (16)
C5—C4—H4	119.8	C1—C14—C13	123.08 (17)
C4—C5—H5	119.9	C13—C14—C9	118.68 (17)
C6—C5—C4	120.29 (17)	C8—C15—H15	117.8
C6—C5—H5	119.9	C16—C15—C8	124.45 (17)
C5—C6—H6	119.1	C16—C15—H15	117.8
C5—C6—C7	121.77 (17)	C15—C16—H16	119.6
C7—C6—H6	119.1	C15—C16—C17	120.83 (18)
C6—C7—C2	117.34 (16)	C17—C16—H16	119.6
C8—C7—C2	120.27 (16)	O1—C17—C16	110.43 (17)
C8—C7—C6	122.32 (17)	O2—C17—C16	126.35 (18)
C7—C8—C9	120.06 (16)	O2—C17—O1	123.22 (18)
C7—C8—C15	121.10 (16)	H18A—C18—H18B	109.5
C9—C8—C15	118.84 (16)	H18A—C18—H18C	109.5
C8—C9—C10	121.88 (17)	H18B—C18—H18C	109.5
C8—C9—C14	120.18 (16)	O1—C18—H18A	109.5
C10—C9—C14	117.93 (16)	O1—C18—H18B	109.5
C9—C10—H10	119.3	O1—C18—H18C	109.5
C11—C10—C9	121.42 (18)	C17—O1—C18	115.31 (16)
			
C1—C2—C3—C4	177.29 (18)	C8—C15—C16—C17	177.77 (17)
C1—C2—C7—C6	−174.75 (17)	C9—C8—C15—C16	128.8 (2)
C1—C2—C7—C8	2.3 (3)	C9—C10—C11—C12	1.3 (3)
C2—C1—C14—C9	2.3 (3)	C10—C9—C14—C1	179.34 (16)
C2—C1—C14—C13	−176.07 (17)	C10—C9—C14—C13	−2.2 (3)
C2—C3—C4—C5	−1.8 (3)	C10—C11—C12—C13	−2.5 (3)
C2—C7—C8—C9	−2.1 (3)	C11—C12—C13—C14	1.3 (3)
C2—C7—C8—C15	177.76 (16)	C12—C13—C14—C1	179.44 (18)
C3—C2—C7—C6	5.1 (2)	C12—C13—C14—C9	1.1 (3)
C3—C2—C7—C8	−177.85 (16)	C14—C1—C2—C3	177.70 (17)
C3—C4—C5—C6	3.6 (3)	C14—C1—C2—C7	−2.4 (3)
C4—C5—C6—C7	−0.9 (3)	C14—C9—C10—C11	1.1 (3)
C5—C6—C7—C2	−3.5 (3)	C15—C8—C9—C10	0.7 (3)
C5—C6—C7—C8	179.56 (18)	C15—C8—C9—C14	−177.88 (16)
C6—C7—C8—C9	174.75 (17)	C15—C16—C17—O1	−173.18 (18)
C6—C7—C8—C15	−5.4 (3)	C15—C16—C17—O2	6.3 (3)
C7—C2—C3—C4	−2.6 (3)	C16—C17—O1—C18	179.04 (17)
C7—C8—C9—C10	−179.43 (17)	O2—C17—O1—C18	−0.5 (3)
C7—C8—C9—C14	2.0 (3)	Br1—C1—C2—C3	−3.1 (2)
C7—C8—C15—C16	−51.1 (3)	Br1—C1—C2—C7	176.78 (13)
C8—C9—C10—C11	−177.55 (18)	Br1—C1—C14—C9	−176.89 (13)
C8—C9—C14—C1	−2.0 (3)	Br1—C1—C14—C13	4.7 (2)
C8—C9—C14—C13	176.41 (17)		

### Hydrogen-bond geometry (Å, º)

Cg1 is the centroid of the C2–C7 ring.

**Table:**

*D*—H···*A*	*D*—H	H···*A*	*D*···*A*	*D*—H···*A*
C15—H15···*Cg*1^i^	0.93	2.91 (2)	3.5055 (19)	123
C16—H16···O2^ii^	0.93	2.40	3.315 (3)	170
C5—H5···O2^iii^	0.93	2.53	3.372 (2)	150

Symmetry codes: (i) *x*, *y*−1, *z*; (ii) *x*, *y*+1, *z*; (iii) *x*, −*y*+1, *z*+1/2.
